# Climate, peace, and conflict—past and present: Bridging insights from historical sciences and contemporary research

**DOI:** 10.1007/s13280-024-02109-1

**Published:** 2025-02-04

**Authors:** Sam White, Dominik Collet, Agustí Alcoberro, Mariano Barriendos, Rudolf Brázdil, Pau Castell, Siyu Chen, Cedric de Coning, Dagomar Degroot, Lukáš Dolák, Stefan Döring, Santiago Gorostiza, Katrin Kleemann, Florian Krampe, Kuan-Hui Lin, Nicolas Maughan, Natália Melo, Barry Molloy, Astrid E. J. Ogilvie, Piling Pai, Qing Pei, Christian Pfister, Silviya Serafimova, Diyang Zhang

**Affiliations:** 1 Department of Political History, Faculty of Social Sciences, University of Helsinki, Snellmaninkatu 14 A, PO Box 54, 00014 Helsinki, Finland; 2 Institute for Archaeology, Conservation and History, University of Oslo, Oslo, Norway; 3 University of Barcelona, Barcelona, Spain; 4 Institut de Diagnòstic Ambiental i Estudis de l’Aigua (IDAEA, CSIC), Barcelona, Spain; 5 Global Change Research Institute, Czech Academy of Sciences, Brno, Czechia; 6 University of Bern, Bern, Switzerland; 7 Norwegian Institute of International Affairs (NUPI), Postboks 7024, St. Olavs Plass, Oslo, 0130 Norway; 8 Environmental History, Georgetown University, Washington, USA; 9 Department of Geography, Masaryk University, Brno, Czechia; 10 Department of Peace and Conflict Research, Uppsala University, Uppsala, Sweden; 11 Research group The Nordic Little Ice Age, Center for Advanced Studies Oslo, Oslo, Norway; 12 Institut d’Història de la Ciència, The Autonomous University of Barcelona, Barcelona, Spain; 13 German Maritime Museum / Leibniz Institute for Maritime History, Bremerhaven, Germany; 14 SIPRI’s Climate Change and Risk Programme, Stockholm, Sweden; 15 Graduate Institute for Sustainability Management and Environmental Education, National Taiwan Normal University, Taipei, Taiwan; 16 CNRS, Aix-Marseille University, Marseille, France; 17 Insitute of Contemporary History, University of Évora, Évora, Portugal; 18 School of Archaeology, UCD, University College Dublin, Dublin, Ireland; 19 Institute of Arctic and Alpine Research (INSTAAR), University of Colorado at Boulder, Boulder, USA; 20 Stefansson Arctic Institute, Akureyri, Iceland; 21 Research Center for Humanities and Social Sciences, Academia Sinica, Taipei, Taiwan; 22 Hong Kong Polytechnic University, Hong Kong, China; 23 Institute of Philosophy and Sociology (BAS), Sofia, Bulgaria; 24 Institute of Environmental Social Science and Geography, Faculty of Environment and Natural Resources, University of Freiburg, Freiburg i. Br., Germany

**Keywords:** Archaeology, Climate change, Conflict, History, Peace, Science communication

## Abstract

Concern has risen that current global warming and more frequent extreme events such as droughts and floods will increase conflict around the world. This concern has spurred both social science research on contemporary climate, peace, and conflict as well as research in the historical sciences on past climate, weather, warfare, and violence. This perspectives article compares these two fields of scholarship and examines how each may benefit the other. It finds significant convergences in methods and insights across contemporary and historical research as well as persistent patterns in causal pathways between climate and conflict. Contemporary climate, peace, and conflict (CPC) research may sharpen methods and causal models for historical researchers. Historical studies, particularly those informed by contemporary research, may elucidate deep origins and long-term effects of climate-related conflicts. For policymakers and the public, history offers comprehensible ways to make sense of complex and contingent linkages and to construct cogent narratives of the past as well as storylines for the future.

## Introduction

Anthropogenic global warming has heightened concerns over links between climate change and violent conflict. In recent decades, a growing number of studies—here termed contemporary climate, peace, and conflict (CPC) research—have investigated current associations between climatic change and variability, weather, or extreme events such as droughts and floods and interpersonal, intergroup, or international violence and peacebuilding. At the same time, a diverse body of research in history, human and physical geography, historical climatology, archeology, and related fields—here termed historical CPC research—has investigated past climatic variability, weather anomalies, and extreme events as factors in warfare, sectarian conflict, and religious persecution during past centuries or millennia. Connections between these current and historical perspectives have not been fully explored, leaving important questions unanswered. Do climate-conflict linkages demonstrate continuities or discontinuities between past and present? Are historical analogies potentially useful or misleading when planning for future scenarios? What might historical CPC research gain from engaging with contemporary studies, and vice versa?

This perspective article aims to address such questions by bridging insights from contemporary and historical research. It builds on findings from a 2023 workshop at the University of Oslo that brought together scholars in diverse fields of peace and conflict studies as well as history, geography, and archeology. Participants found that contemporary research, which benefits from large, high-resolution datasets, indicates ways to improve analysis of historical data and causal links between past climate and conflict. Examining historical cases from the perspective of contemporary CPC research highlighted significant continuities in climate-conflict links between past and present. In light of these continuities, we identified ways that historical examples and perspectives may also bring benefits to contemporary CPC research and decision-making.

In the following sections, this perspectives article first outlines key approaches in historical CPC research as well as issues in evaluating past climate-conflict links (“Historical CPC research: approaches and emerging issues” section). It next reviews developments in contemporary CPC research, emphasizing two key insights: the prevalence of indirect, delayed, and displaced effects on the occurrence of conflict as well as multiple and complex causal pathways linking climatic change, disasters, and conflict (“Contemporary CPC research: two key insights” section).The following “Advancing quantitative historical research on indirect and complex climate-conflict linkages” and “Understanding causal pathways through historical case studies” sections illustrate how both quantitative and qualitative historical research can integrate these insights from contemporary studies to sharpen causal analysis and identify patterns in climate-conflict links. Finally, the “Insights from the past for contemporary CPC research and policy” section of this perspective article discusses possible uses of historical scholarship in contemporary CPC research and policy, including examination of deep origins and long-term effects of conflict, as well as the value of historical narratives in public communication.

## Historical CPC research: Approaches and emerging issues

Scholars have long been interested in the roles of climate and weather in past conflicts, including military strategy and tactics (e.g., Douglas et al. 1978). The growing availability of paleoclimatic data, rising concern over global warming, and popular media discussions of the influence of recent climate change in conflicts such as the Darfur Crisis (2003-) or Syrian civil war (2011-) have also promoted the production and popularity of studies on climate variability and change as potential causes of historical conflicts (Degroot 2018a, b). This section reviews three main approaches to historical CPC research—large-scale quantitative analyses of climate-conflict links, archeological investigations, and qualitative case studies—concluding with an overview of findings and issues.

### Quantitative historical studies

The majority of the most cited studies in historical CPC research analyze quantitative associations between long-term climatic shifts and conflict frequency over large regions (van Bavel et al. 2019). Such studies have emphasized shifts in agricultural output as contributors to social unrest and violence within or between states. For example, studies have tested links between cooling and warfare in preindustrial Europe and China using Granger causality and have found that these links were significant but weakened with growing state capacity and agricultural diversification (Zhang et al. 2007).

To address limitations in historical datasets, studies have focused mainly on associations between temperature or precipitation and the frequency of warfare across large temporal and spatial scales. Long-term temperature and precipitation trends at multi-annual or multi-decadal resolution can be reconstructed from natural proxies such as tree-ring width and density as well as historical documents. Wars—usually defined as conflict between at least two parties, one a state government, that causes over 1000 battle-related deaths—have left a more substantial textual record than other forms of conflict; they are easier to quantify and date; and accessible compendia provide lists of historical wars. Quantitative historical research has focused mainly on literate societies with long histories, where larger and more reliable samples appear more readily available (Degroot et al. 2022).

Approaches used to overcome limited historical data and demonstrate straightforward causal links have also opened these studies to criticisms. Past reviews have found that climate variables considered in these studies may be irrelevant to the occurrence of conflict; some conflict datasets are faulty, particularly for earlier centuries; and the statistical methods can introduce sampling biases and autocorrelation effects (van Bavel et al. 2019, Degroot et al. 2021). In response, researchers in this field have developed new climate and conflict databases and more sophisticated methods of quantitative analysis, which are explored in “Advancing quantitative historical research on indirect and complex climate-conflict linkages” section of this paper.

### Archeological studies

Climate change is a common theme in the archeology of past societal collapse, and studies have often argued that climate change or extreme weather compounded other stressors (Molloy 2023). Although studies have investigated possible climate-conflict links in the early Holocene and even Pleistocene (Crevecoeur et al. 2021), more evidence becomes available with increasing complexity in material culture and land-use regimes during the late Holocene. For example, a reputed 4.2 ka drought event correlates with substantial social changes in several parts of Eurasia, some of which have been associated with conflict (Younes and Bakry 2022).

A 3.2-ka (Late Bronze Age) event in the eastern Mediterranean provides a more detailed case study of archeological CPC research. Tree-ring evidence indicates a long and deep drought in Anatolia 1198–1196 BCE, thought to have destabilized the Hittite empire (Manning et al. 2023). Additional paleoclimate proxies indicate a swing to cooler and more arid conditions from the twelfth to eleventh centuries BCE (Kaniewski et al. 2020). Violent destruction of settlements and raiding around the eastern Mediterranean, identified in the archeological record and in contemporary texts, occurred during these centuries. Conflict accompanied political destabilization, culminating in the political collapse of multiple societies, including the Hittite empire and Mycenaean societies of Greece. Climate change complicated recovery and may be related to violent appropriation of resources and shifts in economic networks revealed in archeological studies (Cline 2021). Disparities in dating the onset and durations of climatic shifts as well as settlement destruction and recovery patterns highlights a need for caution in calibrating and correlating changes in climate and conflict in this setting (Hazell et al. 2022). Further paleoclimate and archeological evidence suggest climate-conflict linkages in prehistoric Europe. For example, abandonment of monumental settlements in the Carpathian Basin and Po Valley coincides with markers of climate change. These co-occur with innovations in warfare and violence that spread from there to the eastern Mediterranean and across Europe (Molloy 2023).

Popular “climate apocalypse” narratives now find little traction in archeological research on these events during the Late Bronze Age. Links between climate change and conflict in specific later periods and places around the world including the Classic Maya, Rapa Nui and Angkor are also debated (Middleton 2017). Cases of climate, conflict, and social change are increasingly analyzed as a combination of underlying social processes and short-term conjunctures better suited to co-analysis alongside climatic variability and change (Middleton 2020).

The temporal and spatial resolution of archeological evidence has been a limiting factor in drawing causal links between climate and conflict (Knapp and Manning 2016). Written evidence is often inadequate to establish causation; and traces of past conflict, including destruction of places or property (material culture) or injuries to persons (osteological), are typically limited and/or ephemeral. Sources for past climate reconstruction range from ice cores, speleothems, palynology, and tree-rings to relative insect abundance, commonly combined with stable isotope or trace element analyses or else new methods such as sedimentary DNA. While some sources (e.g., tree-rings and ice cores) can provide annual resolution, others (including speleothems and pollen) enable reconstructions at only decadal to multicentury resolution. These conditions restrict the attribution of conflicts to climate variability or change. The geographical and temporal coverage of research are also limited. Relationships between climate, conflict, and social change in prehistoric Asia are increasingly being explored (e.g., Laskar and Bohra 2021). However, outside of Egypt, CPC studies of prehistoric Africa remain rare.

### Qualitative case studies

Historians, historical climatologists, and geographers have also used qualitative case studies to understand the role of climate in past conflicts. These have included histories of regional and imperial crises and civil conflict (e.g., Brook 2010); global and comparative studies of crisis periods (e.g., Bauch and Schenk 2019); and more focused studies on the influence of climate variability on outcomes of historical conflicts (e.g., Collet 2023). Most have focused on early modern Europe and East Asia, but research has expanded into other regions such as colonial America.

Studies usually combine high-resolution climate reconstructions from physical and written sources with historical records to create detailed narrative explanations of past conflicts, including political violence, religious persecution, and wars. Climate- or weather-related hazards typically enter these narratives as necessary elements in the sufficient set of factors for the timing, severity, or conduct of these conflicts. For example, White (2011) argues that Ottoman military requisitions set off a large rebellion in Anatolia during the 1590s because the region was already facing exceptional drought, cold, and famine related to the climatic effects of large volcanic eruptions. Degroot (2018a, b) identifies regional climatic conditions as well as the Netherlands’ adaptive capacity as enabling factors in Dutch naval ventures and military successes during the seventeenth century. Collet (2023) contends that a climate anomaly facilitated long-standing designs by hostile neighbors that accelerated geopolitical changes culminating in the First Partition of Poland-Lithuania in 1772. In a wide-ranging study, Parker (2013) emphasizes global climatic change in the history of conflicts across Eurasia known as the “General Crisis of the Seventeenth Century.” Parker’s narrative incorporates numerous political, technological, and social factors, and climate often plays a limited or contingent role in his explanation of specific crisis events (Warde 2015). Nevertheless, global cooling after major volcanic eruptions in the 1640s and 1660s helps make sense of why separate political crises and conflicts across Eurasia occurred synchronously, and Parker’s study demonstrates that extreme weather was often a key condition for exceptionally high mortality during these conflicts.

**Table:** 

**Text Box: Climate and Witchcraft Prosecutions in Early Modern Europe**	
In sixteenth- and seventeenth-century Europe, thousands of individuals, mainly women, were prosecuted and executed for witchcraft. Studies on climate and witchcraft prosecutions, which combine quantitative and qualitative methods, also illustrate challenges in identifying and communicating complex causal links. Behringer (1995) first drew attention to the correlation between cold summers and prosecutions for witchcraft in central Europe during this period, arguing that conditions for waves of witch-hunts were related to subsistence problems, which were in turn related to climate variability. Accusations in witchcraft trials blamed “unnatural” weather such as hailstorms on weather magic. Expanding this argument, Behringer (1999) emphasized that witchcraft accusations emerged from below, specifically from the agrarian communities most affected by extreme weather. Political structures explained different outcomes in different regions. Accusations of witch-hunting did not gain traction in stable territories with complex administrative structures, such as France or the Dutch Republic; however, weak polities in central Europe were more susceptible to witchcraft accusations coming from peasant communities. Subsequent research by Pfister (2007) confirmed the correlation between cold summers and witchcraft prosecutions in central Europe. Pfister concluded that crop failures, cattle disease, price spikes, and epidemics related to climate variability contributed to a search for scapegoats. Nevertheless, difficulties engaging with existing narratives of witchcraft prosecutions focused on gender, class, and religion have limited the influence of climate-based analyses; and most historical research on witchcraft has ignored these findings or received them with skepticism (e.g., Di Simplicio 2013)	

### Findings and issues in historical CPC research

From the perspective of current global warming and conflict, each field of historical CPC research offers significant insights. Among other contributions, quantitative studies demonstrate long-term, large-scale associations between temperature or precipitation and warfare. Archeological studies provide specific investigations of climate-conflict links grounded in physical evidence over millennia of history and demonstrate collaboration across the climate sciences, social sciences, and historical research (Haldon et al. 2018). Qualitative case studies provide detailed narratives of interactions among climatic variability or change, natural hazards, and societies in conflict events (Ljungqvist et al. 2021).

Improvements in methods and data have enabled historical CPC research to formulate more precise, contingent, and defensible claims about climate-conflict links, and this has helped address criticisms of past work for straightforward climatic determinism (Livingstone 2015) and for vague or unsubstantiated claims of climate as contributing factor in conflicts (Selby et al. 2017). Nevertheless, a comparison of these three approaches reveals limits and raises issues. Detailed qualitative case studies highlight the wealth of high-resolution climatic and societal information that may be required to distinguish correlation from causation and to specify the nature of climate-conflict links. Such information is often unavailable in archeological studies and may not be considered for each case of conflict in large-scale quantitative studies based on limited historical datasets. Therefore, quantitative studies based on simple direct associations may overstate climate-conflict links by simplifying or misstating causation—for example, by implying that climatic change was a sufficient cause of conflict incidence when a closer investigation would reveal that climatic change was only a necessary condition for the timing of conflict outbreaks (White and Pei 2020). Yet quantitative CPC studies may also understate climate-conflict links by overlooking more complex causal relationships not easily captured in linear correlations. On the other hand, large-scale quantitative studies raise questions about the representativity of archeological investigations and especially qualitative case studies. Since these studies usually proceed from investigation of exceptional climate events or historical outcomes, they do not necessarily represent typical associations among climate and conflict generalizable to either past or present. Therefore, qualitative studies may overstate climate-conflict links because they emphasize exceptional periods and rarely consider cases where climatic variability or change might have been favorable. Yet individual qualitative case studies may also overstate the specificity and contingency of climate’s role in each outcome by overlooking recurring mechanisms and causal pathways apparent only in large datasets.

In sum, historical CPC research could benefit from models of climate-conflict links that are sufficiently complex to capture the indirect and contingent nature of linkages yet simple enough to represent robust recurring patterns. The following section considers how contemporary CPC research—which can draw on more abundant and precise climate, weather, and societal data than that available for past centuries—may help formulate such models.

## Contemporary CPC research: Two key insights

Contemporary CPC scholarship has evolved significantly during the past two decades. Since the 2010s, numerous studies have identified positive associations between climatic variables or extreme events and the frequency or magnitude of violent conflict at different temporal and spatial scales (e.g., Hsiang et al. 2013). Nevertheless, it remains difficult to establish a consistent, direct link between recent climate change and conflict, particularly interstate war. Past studies encountered problems of sampling bias, lack of contextualization, and uneven geographical coverage (Adams et al. 2018). Moreover, causal linkages have proven complex and contingent, with climate influencing conflict incidence through mediating and interacting variables and under specific conditions (Koubi 2019).

To address these complexities, a growing body of qualitative and quantitative studies from different disciplines now examines how climate change and extreme events can increase the risk of tensions and violence—especially within-country conflict, such as civil wars, social unrest, and communal clashes—in high-risk social, political, and economic contexts (Sakaguchi et al. 2017). Key risk factors identified in the literature include income dependency on agriculture (Vesco et al. 2021) and excluded ethnic groups (Ge et al. 2022), as well as low socioeconomic development or state capacity, intergroup inequality, and a recent history of violent conflict (Mach et al. 2019). Accordingly, the Intergovernmental Panel on Climate Change (IPCC) Sixth Assessment Report finds that climate change impacts can amplify or aggravate tensions within and between states, but the report also emphasizes the mediation of contextual factors as well as political and societal responses (Pörtner et al. 2022). For example, debates on the contribution of drought to the outbreak of the Syrian Civil War have highlighted demographic factors including population growth and rural-to-urban migration; economic factors including unemployment and rising prices; and above all the political factors including dictatorship, repression, and foreign interference (Selby et al. 2017). Although most studies have focused on sources of conflict rather than cooperation, there is evidence that climate-induced scarcity can also create peaceful environments and foster resilient communities and social institutions as well (e.g., Döring and Hall 2023, Ide et al. 2021).

A review of recent contemporary CPC studies by von Uexhall and Buhaug (2021) identifies moderate to significant progress in several research priorities, including the disaggregation of climatic and non-climatic factors to better analyze climate-conflict links at specific scales, studying the diversity of conflict outcomes beyond state-based wars, identifying scope conditions for climate effects on conflicts, and analyzing causal pathways between climate or extreme events and conflict. The following “Indirect, delayed, and displaced impacts” and "Multiple complex pathways” sections of this paper examine two key insights to emerge from these advances in research: the prevalence of indirect, delayed, and displaced effects, and the recurrence of several complex causal pathways linking climate and conflict. The following sections consider how these two insights can help identify convergences between contemporary and historical CPC studies and further historical CPC research. “The Advancing quantitative historical research on indirect and complex climate-conflict linkages” section reviews recent advances in historical databases and methods employed in quantitative historical CPC research and weighs their ability to test for indirect, delayed, and displaced effects as well as complex causal pathways. “The Understanding causal pathways through historical case studies” section re-considers narrative case studies of past climate and conflict in light of mechanisms and causal pathways identified in contemporary CPC research. Although both sections emphasize research based on written records, the insights may apply to quantitative and qualitative analyses in archeological research as well.

In their review, von Uexhall and Buhaug (2021) also find more limited progress in three other research priorities for contemporary CPC studies: understanding the long-term impacts of climatic change (in contrast to effects of short-term variability or extreme events); understanding how political and social responses, including adaptations, to climatic change may influence peace and conflict; and achieving methodological diversity in the field. In the “Insights from the past for contemporary CPC research and policy” section, which considers possible contributions of historical CPC research to contemporary research, we will therefore consider how historical scholarship may advance these priorities.

### Indirect, delayed, and displaced impacts

**Fig. 1:**
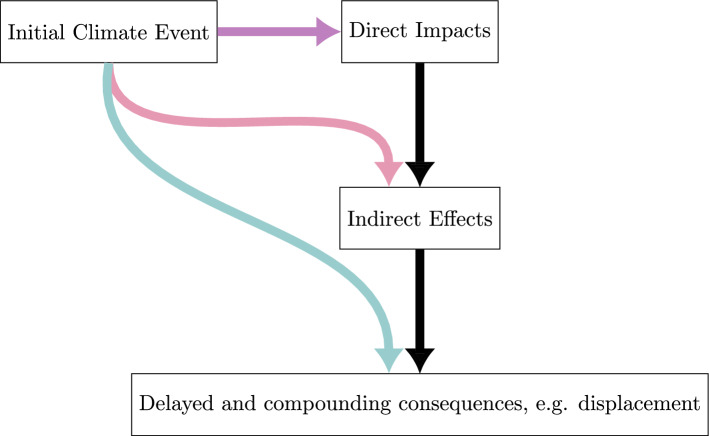
Schema of direct and indirect effects of climatic change and extreme events on the incidence and severity of conflicts

As with other potential recent climate change impacts, climate-conflict risk can be understood as a function of climate-related hazards, exposure to climate effects, changing vulnerabilities, and adaptive or maladaptive responses. As illustrated in Fig. 1, climate-related hazards have both direct effects and simultaneously indirect, delayed, and displaced effects. They may not only trigger conflicts in situations of high exposure and vulnerability but also expose new populations to recent climate change impacts and compound pre-existing vulnerabilities to climate change and disasters (Buhaug et al. 2023). Moreover, recent climate change impacts may interact with conflicts to potentially initiate vicious cycles, trapping societies in a loop of increasing violence and climate-related disasters (Buhaug and von Uexkull 2021).

One important way in which climate-related hazards produce indirect effects and delayed and displaced consequences is through population displacement and migration. Migration in the context of recent climate change and security is multifaceted. It can be a coping strategy, particularly as a planned adaptation to slow-onset climate-related disasters. In other cases, mainly as an involuntary response to sudden-onset disasters, migration may exacerbate or generate vulnerabilities. For example, leaving a water-scarce area may alleviate pressure on local resources but could also heighten competition for water in the destination region. The relationship between climate-related events and migration is often complex and context-specific, depending on environmental and socioeconomic conditions (Kaczan and Orgill-Meyer 2020). Moreover, what begins as a short-term migration strategy—for example, to escape water scarcity—can evolve into a long-term move (Abel et al. 2019). Therefore, researchers and policymakers need to be cautious in their interpretation of available data and broaden metrics of disaster impact beyond just displacement rates. Complicating factors such as climate- and conflict-induced displacement call for more fine-grained data and testing more complex hypotheses.

### Multiple complex pathways

A second theme in current CPC research has been the multiple and complex causal pathways by which recent climate change influences conflict. In most cases, recent climate change interacts with other factors to exacerbate vulnerabilities, grievances, and political tensions (Mach et al. 2019; Koubi 2019). These interactions may be contingent and nonlinear. Assessment of conflict and cooperation outcomes must weigh the context, timing, and spatial distribution of climate change impacts (Beaumont and de Coning 2022). To analyze the spatial and temporal complexity of climate-related security risks, de Coning et al. (2022) have identified five risk dynamics:

1. *Compound risks*, when two or more risk factors interact, resulting in a more complex and heightened risk.
2. *Cascading risks*, when an initial event triggers subsequent risks in a sequential manner, leading to an escalating risk potential.
3. *Emergent risks*, when two or more independent factors, both temporally and spatially, combine to create a new risk that would not exist without the presence of either alone.
4. *Systemic risks*, when multiple risk factors interact in a way that collectively threatens either parts or the entirety of a societal and/or ecological system.
5. *Existential risks*, posing a threat to the existence of a country or culture.

**Fig. 2:**
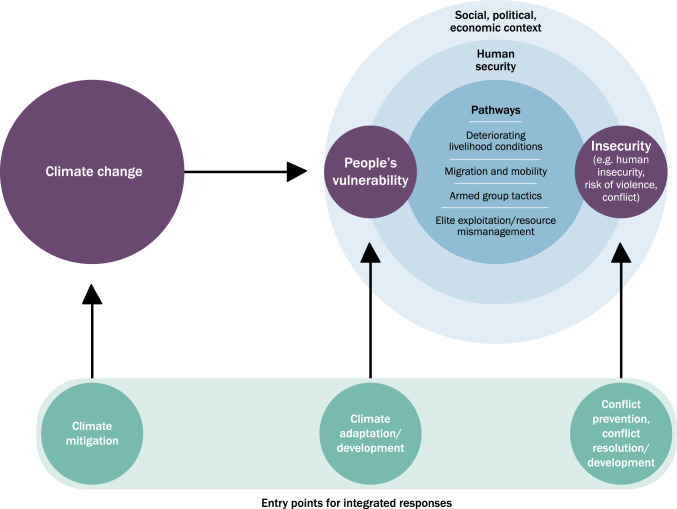
Schema of complex causal pathways between climate change and conflict, based on SIPRI’s Climate and Risk Programme model. Source: de Coning et al. 2022

To navigate the complexity of climate change impacts on society, core elements need to be identified. Figure 2, based on research by SIPRI’s Climate and Risk Programme (de Coning et al. 2022), outlines the relationship by identifying “climate change,” “people's vulnerability,” and “insecurity” as the three core variables to consider within a given context.

Mobjörk et al. (2020) further specify that the mechanisms linking climate change and conflict can be summarized in four pathways that explain different ways that climate-related vulnerability leads to physical violence:

1. *Livelihood deterioration*. Climate change undermines livelihoods, increasing the risk of conflicts by putting pressure on societies or specific populations dependent on sectors such as agriculture and livestock farming.
2. *Migration and mobility*. Climate change impacts contribute to migration and changing mobility patterns, resulting in new conflict risks, for example between incoming groups and residents of destination regions.
3. *Tactical and strategic opportunities for armed actors*. Climate change influences armed actors’ behavior, including their operational readiness and changing power dynamics, which can impact conflict dynamics.
4. *Elite exploitation or mismanagement of grievances*. Climate-related security risks impact governance structures, straining government capacity and resources, leading to weakened institutions, ineffective policies, and potential social unrest.

Factoring in human agency adds an additional layer of complexity (Krampe and de Coning 2022). Recent climate change may influence people’s options, but people choose how to respond to climate-related stressors: climatic variability and change do not wage war, people do. Two households in the same community, or two communities may respond in different ways, depending on their resilience and adaptive capacity, even if their exposure to climate change is similar. Thus, while these models describe patterns in climate-conflict linkages, climate data alone cannot predict conflict in a specific community.

## Advancing quantitative historical research on indirect and complex climate-conflict linkages

As discussed in the “Historical CPC research: approaches and emerging issues” section, quantitative historical CPC studies have responded to past criticism of reductionism and determinism by employing increasingly sophisticated quantitative methods. At the same time, further historical research and a growing availability of high-resolution paleoclimate information have aided in the construction of richer databases of past climates and societies. This section examines how these advances in historical CPC research can potentially identify indirect, delayed, and displaced effects as well as complex pathways between climate and conflict as described in contemporary CPC studies, thereby contributing to a long-term picture and bridging historical and contemporary perspectives. It concludes with a case study employing recent databases of natural hazards and societal information for imperial China.

### Methods in historical quantitative and spatial analysis

Researchers have refined and diversified quantitative methods for identifying and analyzing historical climate-conflict associations from both temporal and spatial aspects. Time-series analysis remains an important approach in demonstrating the significant impacts of climate variability and change, especially in macro-quantitative studies. Established methods include correlation analysis, regression analysis, and the Granger causality test. For more than a decade, the global occurrence of conflict has been examined in direct association with past climate variability (Hsiang et al. 2013). In the meantime, the approaches of time-series analysis on past climate-conflict linkage have been improved to detect and resolve technical issues such as autocorrelation and collinearity (Cappelli et al. 2023).

Superposed epoch analysis (SEA), a method for identifying links between discrete events and continuous time series (Rao et al. 2019), has recently been employed to account for the hysteresis from climatic phenomena to social conflicts. For example, SEA has revealed 1–2-year time lags between volcanic shocks and elevated levels of violence in late-medieval Ireland (Campbell and Ludlow 2021). Nonlinear methods of quantitative analysis have also been developed to address nonlinearity in climate-conflict links. For example, associations between climate variability and nomadic migrations in historical China were interpreted by combining nonlinear econometric methods including threshold regressions, time-varying copula, and nonlinear causality tests (Damette et al. 2020). The boosted regression trees model, a nonlinear machine learning method, contributed to inferring potential causal linkages between positive temperature deviations or precipitation extremes and modern armed conflict at a global scale (Ge et al. 2022).

Bayesian approaches make it possible to integrate information from diverse sources and expertise from multiple fields and also to distinguish meaningful causal relationships from statistical relationships arising from chance, artifacts, or autocorrelation in time series (White et al. 2023). Research in a Bayesian framework has examined causal links between climate variability and societal impacts across different scales and regions. For example, Collard et al. (2021) evaluate effects of temperature and rainfall on conflict counts in the Classic Maya region.

Spatial analyses have explored displaced effects of climate on conflict. The application of geographic characteristics as variables has been applied in research focusing on different spatial scales. In Europe, for example, the spatial extent of cooling was considered as a variable when examining correlations between climatic variability and warfare 900–1999 CE (Lee et al. 2019). Geostatistical analysis on climate-conflict linkage has drawn attention primarily from researchers in China. This includes work demonstrating spatiotemporal relationships between climate and war via standard deviational ellipse (Zhang et al. 2020) and work exploring regional interactions in social responses to drought through network analysis (Zhai et al. 2020).

The types of quantitative analysis discussed in this section have focused mainly on data-rich regions and periods in China and Europe. For other regions, including Africa and the Middle East (e.g., Sofuoğlu et al. 2020), scholars have considered specific pathways by which climate can cause conflicts in modern contexts. These studies explain climate effects on conflicts through mechanisms of migration or economic impact based on multiple quantitative methods including logistic regressions, structural equation modeling (SEM), and cross-sectional augmented Dickey-Fuller (CADF).

### Climate and societal databases for historical research

Quantitative approaches have also built on richer databases of past climates and societies. High-resolution paleoclimate reconstructions cover new regions and centuries at higher resolution than previously available. Important developments include further recovery of early instrumental records, analysis and indexing of pre-instrumental weather descriptions, and tree-ring-based gridded drought reconstructions for North America and much of Europe and Asia. More recently, Burgdorf et al. (2023) have compiled a global documentary climate dataset (DOCU-CLIM) including 621 time series providing historical information of long-term temperature, precipitation and wind variations. Reanalyses of paleoclimate data incorporating improved climate modeling techniques, a broader proxy record, and better integration of early instrumental data have also produced datasets with higher coverage and homogeneity particularly for data-scarce regions and past centuries (Valler et al. 2024) (Table 1).

**Table 1:** Databases of past climate and natural hazards for historical CPC research

Name of database	Description	Source
NOAA World Data Center for Paleoclimatology	Global database of paleoclimate data generated from natural and social archives across local to global spatial scales and decadal to multi-millennial temporal scales	www.ncdc.noaa.gov/paleo
Tambora.org	Database of worldwide data on climatic parameters such as temperature, precipitation, storms, and floods from man-made sources	https://tambora.org/
Euro-Climhist	Database of historical weather events and natural hazards in Europe, including descriptive data, instrumental data, biophysical proxy data, and indices	https://www.euroclimhist.unibe.ch/
Swedish Climate History Database 1500–1870	Database extracting weather-related records, mostly in daily resolution, from historical documents in northern Europe ca. 1500–1870	https://snd.gu.se/sv/catalogue/dataset/snd1216-1/1
East Asian Paleoenvironmental Science Database	Database of paleoclimate, paleoenvironmental, real-time observation, and simulation data from East Asia decadal up to millennial timescales	http://paleodata.ieecas.cn/
REACHES	Digitizes and categorizes meteorology and climate-related records from historical documents of China in the last 3000 years	https://reaches.rcec.sinica.edu.tw/
Historical Weather Database on the Web	Collects, classifies, and visualizes daily weather information in Japan back to the seventeenth century	http://tk2-202-10627.vs.sakura.ne.jp/
INPRO	Database compiling information on pro pluvia rogation ceremonies (a proxy of agricultural drought) across 11 countries in Europe, America, and Asia 1333–1949	http://inpro.unizar.es/
HCLIM	Global climate dataset of monthly instrumental series pre-1890	https://doi.org/10.1594/PANGAEA.940724

Rising availability and quality of past societal data have been equally important for improving analyses of historical climate-conflict links and particularly for testing indirect effects and complex patterns. Researchers have created or improved databases for key societal variables including harvests, prices, and migrations, as well as historical records of disasters and violence (see Table 2). For example, the Historical Social Conflict Database (HiSCoD) is a large dataset in development containing ~ 21 k episodes of social conflict ca. 1000–1900 CE in Europe and parts of North America, Turkey, and Africa (Chambru and Maneuvrier-Hervieu 2022). The HiSCoD project aims to analyze possible causal linkages between climate variability and change and past European conflicts, using both climatic data and additional variables influenced by climate. The database has collected existing information from a wide range of studies and sources, including detailed analyses of social conflict during the French Revolution. HiSCoD categorizes past conflicts into 10 types: food riot, tax riot, religious conflict, conflict with local or national authorities, feudal conflict, slave revolt, political conflict, labor conflict and banditry, and other forms of conflict. This overcomes issues of inconsistency, lack of detail, and lack of historical context that have generated problems in previous research.

**Table 2:** Databases of societal information for historical CPC research

Name of database	Description	Source
HiSCoD	Social conflicts mostly in Europe from the Middle Ages to late nineteenth century	https://www.unicaen.fr/hiscod/accueil.html
IISH Data Collection	Data platform containing a large number of worldwide datasets on demographic, social, and economic history	https://datasets.iisg.amsterdam/dataverse/IISH
Clio Infra	Data platform containing interconnected databases of worldwide data on social, economic, and institutional indicators for the past 500 years	https://clio-infra.eu/
Allen-Unger Global Commodity Prices Database	Global database of Commodity price series from the late Middle Ages to the early twentieth century	http://www.gcpdb.info/
Historical Database of Scanian Agriculture	Micro-level database containing information on farm production in southern Sweden 1702–1881	https://portal.research.lu.se/en/publications/historical-database-of-scanian-agriculture-version-30
SEDD (Scanian Economic Demographic Database)	Database of demographic and socioeconomic information in Scania 1646–1967	https://www.lusem.lu.se/organisation/research-centres/centre-economic-demography/cedpop-databases-ced/scanian-economic-demographic-database-sedd
Database of Japan's Early Modern Economy	Daily information on weather and prices in Osaka mid-18th to late-nineteenth centuries	https://www.rieb.kobe-u.ac.jp/project/kinsei-db/index.html
Asian Historical Statistics Database	Historical economic statistics of Asian areas back to the late nineteenth century	https://hi-stat.ier.hit-u.ac.jp/english/research/database/ashstat.html
Qing Dynasty Food Price Database	Monthly grain price data during the Qing Dynasty (1644–1911)	https://mhdb.mh.sinica.edu.tw/foodprice/

The chronological and geographical reach of both climate and societal data remain uneven, with greater coverage of early modern Europe and China than other regions and periods. For some regions, including the Arab world, the Americas, and Africa, more efforts in data compilation, classification and digitalization from local archives are necessary. Natural archives of climate tend to have larger spatial coverage and a longer time span, while human records and textual documents particularly, are often more localized and discontinuous. Nevertheless, the latter may have very high temporal and spatial resolution especially valuable in micro-level studies.

### Case study: The REACHES and SIER databases and climate-conflict links in late imperial China (1776–1851)

The early modern Chinese state kept exceptionally rich records concerning weather and climate, especially extremes affecting crops, as well as abundant socioeconomic information including taxes, grain yields, population movements, epidemics, and episodes of violence and conflict. Since the 1960s, Chinese scholars have gathered, coded, and indexed this information in specialized atlases and chronologies (e.g., Liang 2008) employed in many of the studies described above.

First established in 2018 and regularly updated since, the REACHES database (Wang et al. 2018) provides a searchable online platform of historical Chinese records. REACHES includes a range of climate- and weather-related phenomena, with time and location information of all occurrences encoded and georeferenced in the database. It enables multivariate temporal and spatial analysis for phenomena of interest at annual, seasonal, and sometimes daily or weekly resolution. The Social Impact Event Records (SIER) database, started in 2020, supports a corresponding analysis for past societal impacts associated with climate and weather. The project collects historical documents in three categories—population and economy, epidemics, and warfare—each from different sources of archival data and source books. All information is digitized, encoded, and georeferenced in SIER, using a relational database system compatible with REACHES to support cross-database analysis.

**Fig. 3:**
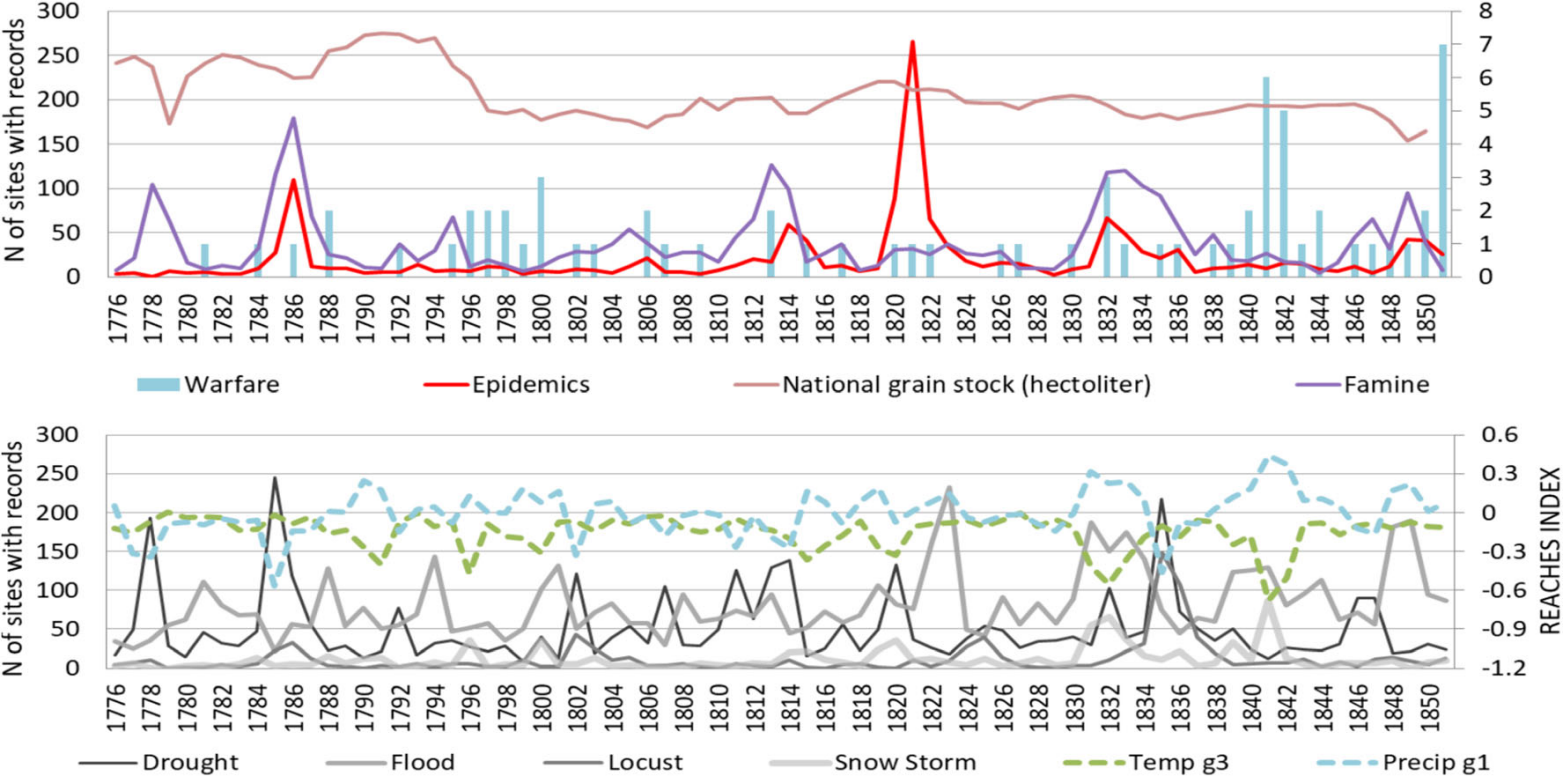
Time series of climate and society variables in China 1776–1851. The upper panel shows socioeconomic variables extracted from the SIER database, including warfare (blue bar), epidemics (red curve), national grain stock (light red), and famine (purple). Note that national grain stock (hectoliter) is calibrated to 0–300 (original value * 0.000006 (= 6.E−06)). The lower panel shows climate variables from the REACHES database: reconstructed temperature index (dashed green), precipitation index (dashed blue), as well as droughts, floods, locust swarms, and snowstorms

The abundance and precision of combined climatological and societal data in REACHES and SIER enables exceptionally complex and fine-grained statistical and spatial analyses of past climate-conflict links. As a case study, the period 1776–1851 contains reliable population data and also contains numerous conflict events, which led up to the Taiping Tien Guo rebellion ( ). As shown in Fig. 3, grain stocks peaked in the late 18th but fell during conflicts in the 1790s. Famine and epidemics followed, particularly during the cold decade of the 1810s.

**Fig. 4:**
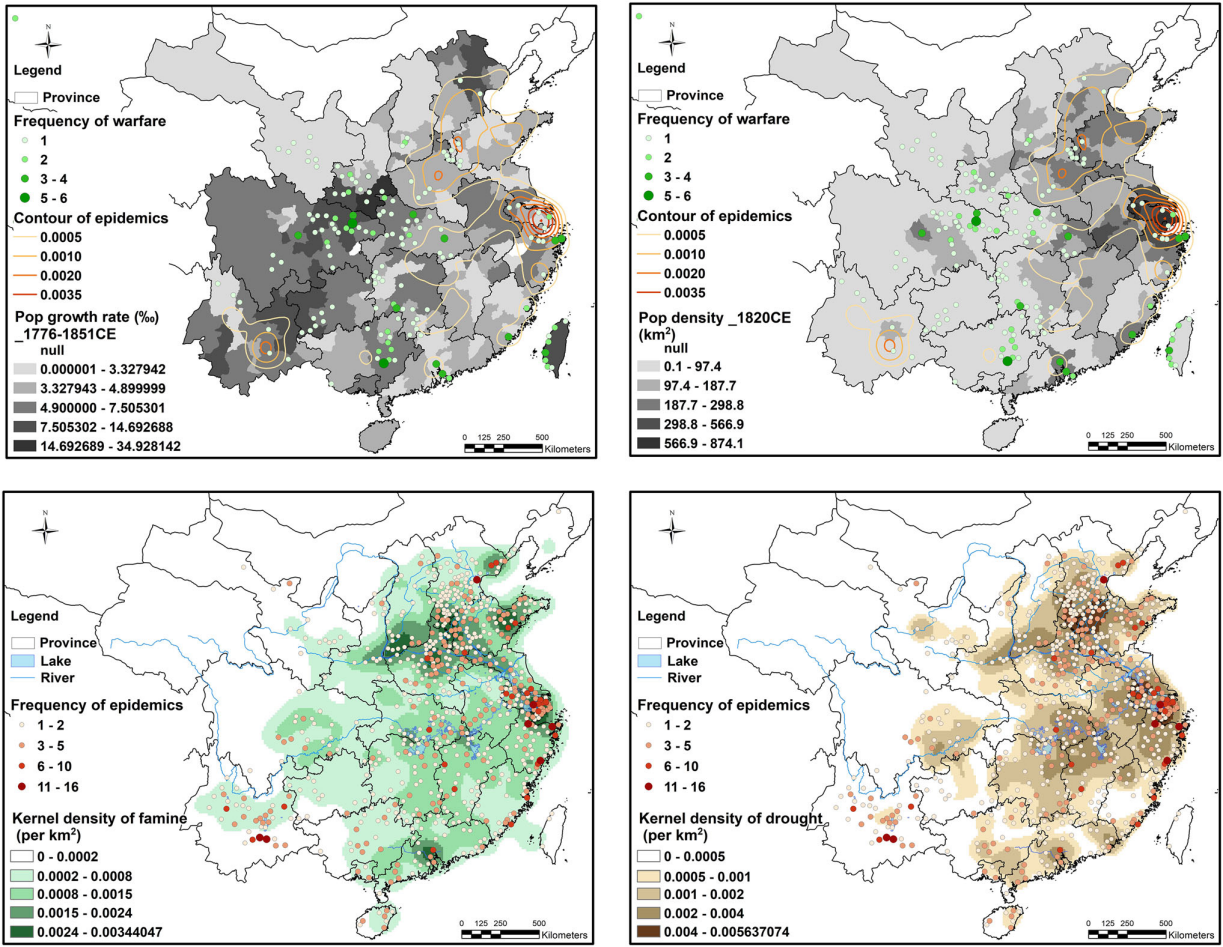
Spatial analysis of natural hazards and societal variables from the REACHES and SIER databases. The upper maps display the associations between epidemics and drought (top left) and epidemics and famine (top right) during 1776–1851. The lower maps display the associations in the same period between warfare and population density (bottom left) and warfare and population growth rates (bottom right). The latter indicates migration from eastern regions with high population density and epidemic disease rates into southwestern China, where conflict erupted

Spatial analysis reveals further patterns not apparent in the time series alone (Fig. 4). Droughts in this period centered on northern China, but famine extended to other regions, including the southwestern province of Yunnan. Epidemics clustered in the most densely populated regions in eastern China, but conflicts clustered in low-density regions with high population growth especially in Shaanxi, Sichuan, Hubei, and Guangxi, which received a high proportion of poor, unemployed, and homeless migrants. Thus, historical climate-conflict links demonstrate similar indirect, delayed, and displaced patterns of impacts as found in some modern episodes through mechanisms of migration as well as shifting exposure and vulnerability (see “Contemporary CPC research: two key insights” section).

## Understanding causal pathways through historical case studies

As described in the “Historical CPC research: approaches and emerging issues” section, historical research has also employed detailed qualitative case studies to narrate causal connections between climate and past conflicts. However, the focus of case studies on exceptional climatic conditions and historical outcomes may overstate climate-conflict links or overlook recurring causal pathways and mechanisms. Using two examples from the “general crisis” period (see “Qualitative case studies” section), this section examines climate-conflict links in historical case studies in light of findings from current CPC research. While rooted in specific historical circumstances, these case studies illustrate broad continuities between past and present climate-conflict links, particularly recurring casual pathways rooted in livelihood deterioration, population displacement, tactical opportunities for armed actors, and elite mismanagement of grievances as described in the “Contemporary CPC research: two key insights” section.

### Central Europe during the Thirty Years’ War: Cascading effects of climate on conflict

The Thirty Years’ War (1618–1648) was one of the most destructive wars in European history, resulting in the loss of roughly one-third of the population within present-day Germany as well as enduring shifts in European geopolitics (Wilson 2009). Although the onset of the Thirty Years’ War was not directly related to climate, the war occurred during a period of exceptional cooling, highlighting possible climate-conflict links. A recent review (Brázdil et al. 2023) explores climate’s multiple and indirect roles in exacerbating and perpetuating conflict by increasing exposure and vulnerabilities to hazards and by creating opportunities exploited by belligerents. Climate anomalies and weather extremes that characterized the period often coincided with other societal and economic stressors, which amplified their negative impacts. Extremely cold winters during the Thirty Years’ War could directly influence military operations—i.e., facilitating river crossings, such as when Hungarians traversed the frozen Morava River in February 1621. Fatal frost events could devastate armies in the field, such as in Bohemia and Moravia in February 1624, December 1626, and the winter of 1635 (see also Degroot 2018a, b).

More consequential, however, were delayed and indirect effects, such as repeated harvest failures. Dry spells, particularly in the 1630s, reduced grain yields, halted the operation of water mills, and increased the frequency of forest fires. At other times, heavy rains and floods complicated grain harvests and storage. Resulting hunger and poor hygienic conditions rendered populations more susceptible to contagious diseases such as plague and dysentery, which were exacerbated by the movement of troops across central Europe. These indirect impacts in turn paved the way for delayed and compounded consequences. Under-resourced armies resorted to quartering orders, plunder, and violence against civilians. Populations weakened by warfare, hunger, and disease were unable to cultivate fields and produce food even after weather conditions improved. These cascading effects could lead to a downward spiral, generating extended and displaced impacts. Conversely, regions less affected by local war events and at a distance from troop movements were better able to cope with negative effects of climate and weather (Brázdil et al. 2023).

### Catalonia 1580–1650: Disasters, political tensions, and violence

**Fig. 5:**
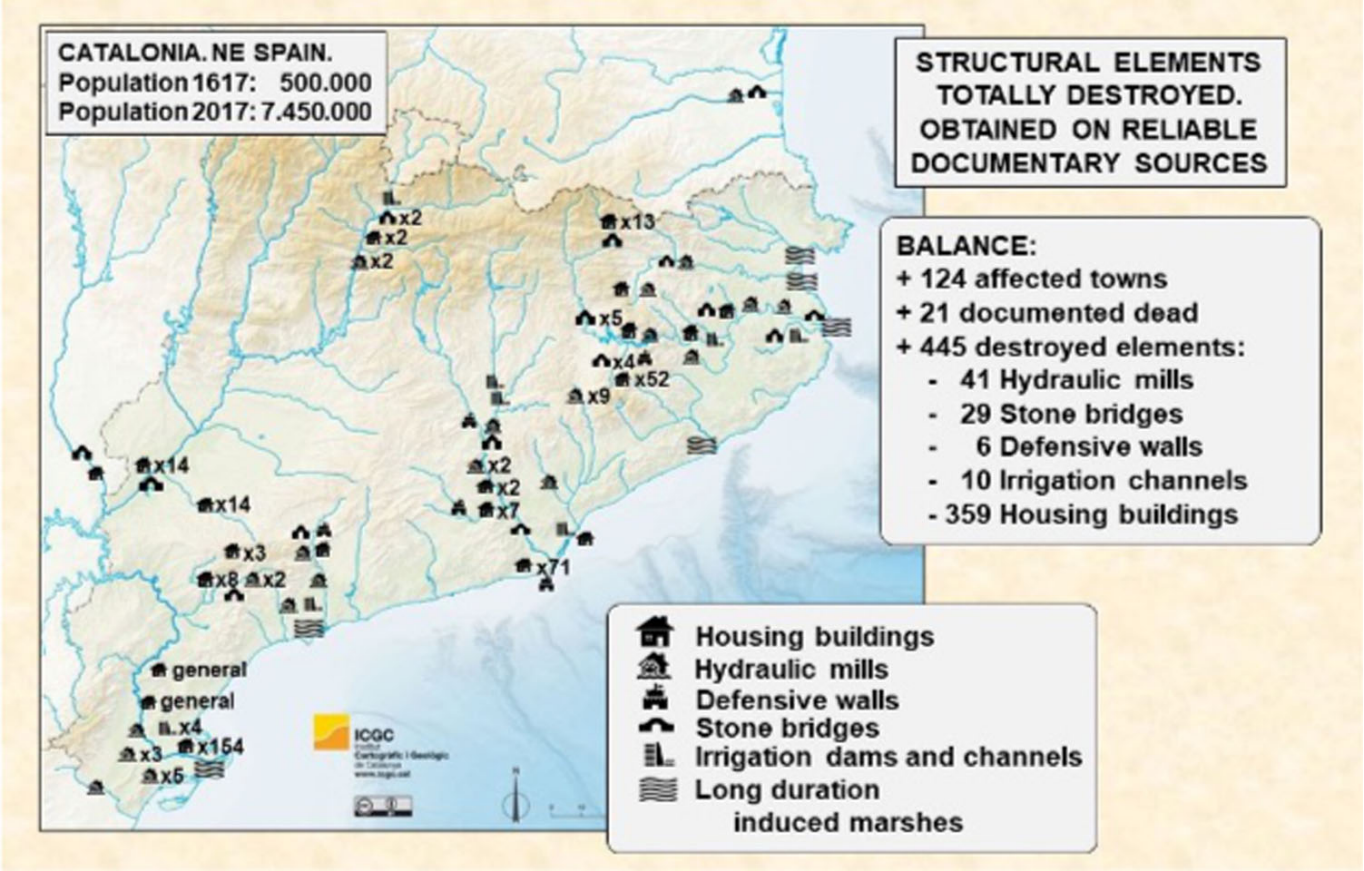
Major flood events and structural damage in Catalonia, November 1617. Source: D. Pino et al. 2018 “Major flood events reconstruction from a multi-proxy approach. The case study of November 1617 flood event in the Mediterranean Basins of Iberian Peninsula”, *Geophysical Research Abstracts*, 20, EGU2018-10386, ISSN 1029–7006

Similar graduated pathways can be observed in Catalonia. The region experienced a period of exceptional flooding and drought from the late sixteenth to the mid-seventeenth century, which can be reconstructed from both documentary evidence and natural proxies (e.g., Barriendos and Martin-Vide 1998). Peak years of flooding from the 1580s to 1610s affected large parts of Iberia (Barriendos et al. 2019), including an exceptional rainfall event on 2–6 November 1617, which caused extraordinary floods throughout Catalonia with extensive structural damage (Thorndycraft et al. 2006) (Fig. 5).

The period has been analyzed for two kinds of linkages between climate and conflict. First, as discussed by Grau-Satorras et al. (2016), droughts intensified pre-existing conflicts over access to water, and these disputes exacerbated intra- and inter-regional contests for control of resources, especially during the late 1620s (Simon i Tarrés, 1992). Plague and conflict in France and northern Italy related to the Thirty Years’ War also interrupted grain imports. In spring 1631, protests over grain prices and scarce bread turned into violent riots. In response, the Barcelona city council (Consell de Cent) assumed full control of bread production and implemented a centralized rationing system (Simon i Tarrés 1992).

Moreover, these direct actions initiated a range of delayed responses. Over time, municipal controls instituted to calm popular violence became a subject of further political conflict (Grau-Satorras et al. 2021). Disputes over water rights between Barcelona’s city government and the cathedral chapter ignited in 1634, when the municipality tried to enforce regulations to control the production and distribution of bread. The cathedral’s chapter responded by arresting a city officer, and in response, the city cut the water supply to the cathedral. This decision led to the excommunication of all the members of the city government. The conflicting parties exploited drought as a tool in an ongoing political contest, aggravating minor quarrels into major institutional disputes, which persisted over the following years (Gorostiza et al. 2021).

**Fig. 6:**
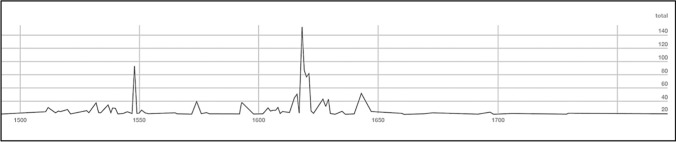
Cases of witchcraft accusations in Catalonia. Source: Castell (2022) and *Atles de la cacera de bruixes* (2021)

Second, the period of floods and droughts coincided with a large and well-documented phase of witchcraft accusations in Catalonia. Over 1000 executions for “witchcraft” have been documented in early modern Catalonia, mostly during the early seventeenth century (Fig. 6), as recently cataloged by Castell (2022) and displayed in an online database (Atles de la cacera de bruixes, 2021).

Witchcraft prosecutions, including accusations of weather manipulation, peaked following the destructive 1617 flood and crop failures of 1618 (Kamen 1993). Alcoberro (2024) has found that weather manipulation was often the only accusation that started legal cases against women. As early as 1600, local courts used torture to extract confessions about the conjuring of hailstorms. During the following years, and particularly in the aftermath of the 1617 floods, these confessions led to new waves of arrests, which spread persecution throughout the territory.

These witchcraft prosecutions may be analyzed as a case of elite mismanagement of popular grievances resulting in violent scapegoating. Prosecutions targeted predominantly women in marginal situations. More centralized kingdoms within the Spanish monarchy saw almost no witchcraft-related death penalties in this period; however, in Catalonia during this period of disasters, the conflicts escalated into a contest between popular agency and the authority of the church, as highlighted by Behringer (1999). Figures in the Catholic Church in Catalonia opposed the witch-hunts and challenged accusations of weather manipulation, and by 1630 royal intervention halted the witch-hunting phenomenon (Alcoberro 2014). Nonetheless, hundreds of women were killed in the meantime, and popular belief in the ability of witches to manipulate weather persisted for centuries (Castell 2022).

## Insights from the past for contemporary CPC research and policy

The significant continuities between past and present climate-conflict links identified in this perspectives article indicate that historical studies may also provide valuable perspectives on contemporary climate change and conflict and insights that complement those of contemporary CPC research. In particular, this section considers how historical scholarship may advance research priorities raised in the “Contemporary CPC research: two key insights” section, including long-term impacts of climatic change on peace and conflict, consequences of political and social response to climatic change, and methodological diversity.

As discussed in the “Contemporary CPC research: two key insights” section, contemporary CPC studies have found that climatic variability and extreme events increase the risk of tensions and violence particularly in high-risk social, political, and economic contexts. Historical research can elucidate long-term origins and development of these contexts as well as interactions between underlying structural vulnerabilities and climate-related hazards. For example, historical research reviewed for this study indicated a pattern of climate-related “slow violence” leading up to outbreaks of conflict. As discussed in Nixon (2013), slow violence refers to less flagrant or visible forms of violence, primarily directed at the poor and vulnerable, and includes the denial of traditional means of adaptation or coping during environmental hazards. Harsh winters occurring with greater frequency throughout the early modern “Little Ice Age” constituted a major risk for poorer populations, who relied on access to charity and shared resources for survival; therefore increased tax or rent demands and restrictions on mobility or access to commons during these winters constituted a deadly form of slow violence (Collet 2023). In a case study of late Ottoman Kurdistan, for instance, Pehlivan (2024) finds that restrictions on relief aid and credit to pastoral populations following major winter storms and loss of livestock during the 1880s–1890s created desperate conditions among Kurdish pastoralists, facilitated recruitment into irregular military groups, and fueled the intercommunal violence that led up to massacres of Armenian populations in eastern Anatolia and later the Armenian genocide.

Historical research also opens a longer-term perspective on the outcomes of conflicts triggered or exacerbated by climatic variability or change. Investigating events that have unfolded for generations or centuries may contribute to understanding when or whether climate-related conflicts have had particularly severe or enduring consequences compared to other conflicts, and what has made societies more or less resilient to combined impacts of conflict and climatic variability or change. For example, research on the General Crisis of the Seventeenth Century discussed in the “Historical CPC research: approaches and emerging issues” section also examines how greater environmental and social vulnerabilities in Mediterranean regions contributed to an earlier onset of political turmoil and slower economic and demographic recovery from conflict compared to countries in northwestern Europe with more diversified agriculture and economies (White 2011; Parker 2013; Degroot 2018a, b). On the other hand, a historical study of the Near East from the 6th to seventeenth centuries finds that political strategies to achieve short-term resilience following environmental shocks, including climate variability, sometimes shifted impacts onto the poor and vulnerable, setting the stage for future conflict (Izdebski et al. 2018).

In terms of methods, the limited evidence available to historical CPC research has often encouraged ingenuity in the use and combination of evidence. Scholars have learned to integrate qualitative and quantitative data as well as physical and written records in ways that may prove useful to contemporary research as well. Whereas much contemporary CPC research has become increasingly specialized with the growth of the field, historical CPC research projects have typically become larger and more interdisciplinary, sparking productive discussions about consilience across the humanities, social, and natural sciences (Haldon et al. 2018).

Furthermore, as discussed in IPCC WG II, drawing lessons from climate impact research involves not only finding present associations but also developing compelling storylines and scenarios (Pörtner et al. 2022). Historical narratives of climate, peace, and conflict may prove especially significant given the emphasis within climate change communications on physical sciences and quantitative projections. For example, between 1992 and 2018 over 300 museum exhibitions worldwide addressed climate change and the Anthropocene. Roughly half were led by science-related institutions and focused on climate science, climate literacy, and social awareness through science communication (Melo 2023), while most of the rest focused on contemporary art. Efforts to address climate change and foster societal agency in exhibitions such as these may meet resistance from audiences’ prevailing perceptions. These include perceptions of climate change as a geographically remote or abstract risk, uncertainties about and obstacles to decision-making, as well as personal and political ideologies (Kahan 2012). Exhibitions focused on communicating physical science methods and data also tend to look from the deep past (e.g., ice ages) into a future of disastrous global warming (Melo 2023). Previous research has found, however, that most individuals comprehend the world through narratives and images and that using real-life experiences and everyday analogies can help people to cope with risk (Corner et al. 2015). Relatable historical narratives—particularly place-based narratives—reduce the psychological distance between the public and climate risk and emphasize human agency. They reduce the uncertainty factor, especially among skeptical and conservative audiences, by focusing on imminent risks of and past responses to climate-related hazards.

The recent publication and exhibition of a seventeenth-century book in Barcelona illustrate the potential of local history and heritage to transmit cultural memory about climate-related hazards and adaptations and conflict risk. The “Book of Fountains” (1650) was compiled during a period of drought, flooding, and social conflict in Catalonia in the first half of the seventeenth century (see “Catalonia 1580–1650: disasters, political tensions, and violence” section). It presents a city officer’s management of urban water supplies and encapsulates institutional learning during three decades of severe water stress. It codifies knowledge about water rights, distribution, and maintenance, and constitutes a mechanism to store and transmit key knowledge to cope better with environmental stress. In addition, the “Book of Fountains” served a political purpose, reinforcing the city government’s legal claims over the management of urban water supply and heading off future disputes over water rights. Underlining its significance, the municipal government decreed that the book had to be kept within the city for future generations. Recognizing its renewed significance in the current context of global warming, extreme floods and severe dry spells in the region, the city archive has supported the publication of the “Book of Fountains” and has planned an exhibition featuring it (Martí Escayol et al. 2022).

## Conclusion

Bridging insights from current and historical CPC scholarship can benefit both fields of study and enhance understanding of climate-conflict links. As discussed in this perspective article, current research has demonstrated the prevalence of indirect, delayed, and displaced effects as well as complex recurring pathways between climate change and conflict. These current insights can guide the study of past climate and conflict, especially as historical research acquires richer, higher-resolution information on past climates and societies. Employing new databases, new techniques of causal and spatial analysis, and models grounded in current research, historical scholarship can address previous issues studies while illuminating very long-term processes and outcomes difficult to analyze in contemporary research. They also provide the narratives necessary to connect current predictions to lived experiences of climate-related conflicts and slow violence.

While the examples here highlight continuities in climate-conflict pathways between past and present, the discontinuities and divergences also call for further interdisciplinary research. Moreover, both the contemporary and historical research considered here has focused on sources of violence rather than sources of cooperation and peace. This review particularly draws attention to the need for more histories of states and societies that found ways to draw benefits from climatic change and situations where climatic variability or extreme events provided opportunities for building peace. The agenda and approach discussed here can underpin future research on historical conditions that may have encouraged more inclusive and cooperative alternatives. It can also remind policymakers of the scope for human agency apparent in historical variance and plurality. Lastly, it can provide the narratives necessary to envision and enact future change.
